# Value of hospital antimicrobial stewardship programs [ASPs]: a systematic review

**DOI:** 10.1186/s13756-019-0471-0

**Published:** 2019-02-12

**Authors:** Dilip Nathwani, Della Varghese, Jennifer Stephens, Wajeeha Ansari, Stephan Martin, Claudie Charbonneau

**Affiliations:** 1 Ninewells Hospital and Medical School, Dundee, DD19SY UK; 2 Pharmerit International, 4350 East West Highway, Suite 1100, Bethesda, MD 20184 USA; 3 Pfizer, New York City, NY USA; 4 Pfizer, Paris, France

**Keywords:** Antibiotic stewardship program, Antimicrobial resistance, Economic evaluation, Antimicrobial stewardship

## Abstract

**Background:**

Hospital antimicrobial stewardship programs (ASPs) aim to promote judicious use of antimicrobials to combat antimicrobial resistance. For ASPs to be developed, adopted, and implemented, an economic value assessment is essential. Few studies demonstrate the cost-effectiveness of ASPs. This systematic review aimed to evaluate the economic and clinical impact of ASPs.

**Methods:**

An update to the Dik et al. systematic review (2000–2014) was conducted on EMBASE and Medline using PRISMA guidelines. The updated search was limited to primary research studies in English (30 September 2014–31 December 2017) that evaluated patient and/or economic outcomes after implementation of hospital ASPs including length of stay (LOS), antimicrobial use, and total (including operational and implementation) costs.

**Results:**

One hundred forty-six studies meeting inclusion criteria were included. The majority of these studies were conducted within the last 5 years in North America (49%), Europe (25%), and Asia (14%), with few studies conducted in Africa (3%), South America (3%), and Australia (3%). Most studies were conducted in hospitals with 500–1000 beds and evaluated LOS and change in antibiotic expenditure, the majority of which showed a decrease in LOS (85%) and antibiotic expenditure (92%). The mean cost-savings varied by hospital size and region after implementation of ASPs. Average cost savings in US studies were $732 per patient (range: $2.50 to $2640), with similar trends exhibited in European studies. The key driver of cost savings was from reduction in LOS. Savings were higher among hospitals with comprehensive ASPs which included therapy review and antibiotic restrictions.

**Conclusions:**

Our data indicates that hospital ASPs have significant value with beneficial clinical and economic impacts. More robust published data is required in terms of implementation, LOS, and overall costs so that decision-makers can make a stronger case for investing in ASPs, considering competing priorities. Such data on ASPs in lower- and middle-income countries is limited and requires urgent attention.

## Background

Antimicrobial resistance (AMR) is a global problem threatening not only public health but also economic development and security. Globally, AMR has the potential to cause 10 million deaths by 2050 based on high-level scenarios [1]. The World Bank estimates that there will be up to one trillion US dollars global increases in healthcare costs by 2050 due to AMR [2].

AMR can stem from inappropriate antibiotic use which can include overuse, misuse, underuse or abuse of antibiotics [3]. Rate of antimicrobial misuse in hospitals, including failure to de-escalate and overprescription of broad spectrum antibiotics, has remain unchanged at 50%. Antimicrobial stewardship programs (ASPs) are one way to address inappropriate antimicrobial use and AMR.

The goals of ASPs are to improve patient outcomes and safety and to reduce AMR and healthcare costs by promoting judicious use of antibiotics. Some core elements identified in successful ASPs include leadership commitment, prescriber accountability, drug expertise and education of clinicians and patients, among others [4, 5], ASPs may require additional resources, such as hospital personnel and equipment, in order to be effective and be sustainable. As such, the upfront costs associated with these additional resources can be a potential barrier to individuals who have not yet implemented an ASP. With the growing importance of measuring the impact of ASPs and health economic evaluations, there has been an increasing number of studies that have evaluated the clinical and economic impact of ASPs in the last few years.

A systematic review was conducted by Dik et al. to evaluate methods of published economic evaluations of hospital ASP studies from January 2000 to November 2014 [6]. The authors identified 99 studies, the majority of which were conducted in North America and Europe. Although distinct types of stewardship interventions were evaluated, “Therapy evaluation, review and/or feedback” was the most common. As the primary objective of this review was to evaluate only the quality of these studies, no synthesis of results was performed or aggregated results reported. Given the growing body of literature, an update of the Dik review including a complete summary of the outcomes from these studies was still needed. Therefore, our objective was to conduct a systematic review to update, evaluate, and broadly summarize the clinical and economic impact of ASPs using the Dik et al. framework.

## Materials and methods

### Literature search

This systematic review followed the PRISMA (Preferred Reporting Items for Systematic Reviews and Meta-Analyses) protocol to synthesize the results related to key outcomes from all the individual studies and define an overall value framework for ASPs. The previously identified systematic literature review by Dik et al. (January 2000–November 2014) was used to provide the framework for this review [6].

An updated search to the Dik et al. systematic review was performed within the EMBASE and MEDLINE databases to further include studies after 30 September 2014 up to 31 December 2017, using the following search strings: “antimicrobial stewardship,” “antimicrobial management,” “antimicrobial prescribing intervention,” and “antimicrobial program intervention.” All strings were in combination with the words “cost(s),” “financial,” “economic,” “dollar” or “euro,” or the respective symbols for the latter two. Two authors independently reviewed the retrieved abstracts for their eligibility followed by full-text screening of selected articles. Studies identified through a handsearching process were also included.

### Study selection

**Fig. 1:**
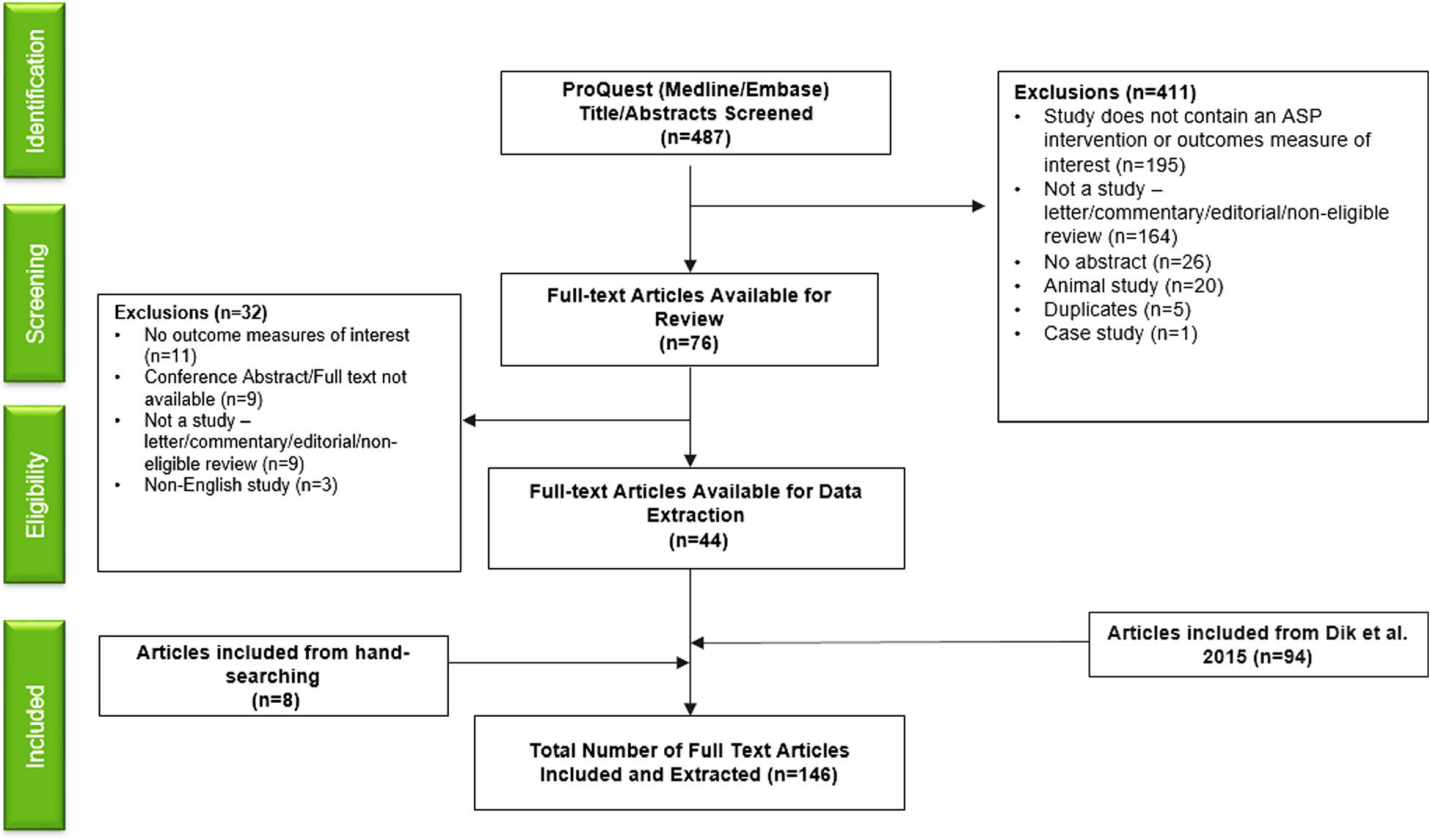
PRISMA chart of the search method

Primary research studies in English that discussed a hospital intervention and key patient and/or economic outcomes, identified previously by Dik et al., were included in this analysis. Our review expanded the search to also include studies published through December 2017 (Fig. 1). Studies that did not contain an ASP intervention, measure any key outcomes, or that were conducted in an animal population were excluded. All inclusion and exclusion criteria were established prior to the review.

### Data extraction

Information collected from each study included publication year, region and country, study objective, study design, setting, hospital size (number of beds), number of participants/patients included, inclusion and exclusion criteria for study data. Information collected about the intervention included type of intervention based on categories reported in Dik et al., description of the ASPs (stewards and their role, components, duration, primary outcomes), outcomes measured and their corresponding results, and the conclusion of the study findings.

The main outcomes of interest were antimicrobial, patient and economic outcomes. Antimicrobial outcomes included antibiotic usage and resistance. Length of stay (LOS), mortality rate, and readmission rates at varying timepoints were the main patient outcomes of interest. Key economic outcomes were costs associated with antimicrobials, LOS, and implementation and operation of ASPs. Outcomes of interest were separated into statistically significant results, non- significant results or results of unknown significance. The majority of the studies that measured significance reported *p*-values with only one study reporting odds ratios [7]. Confidence intervals were utilized in a few studies but also alongside *p*-values. In this review, we therefore only examined significance based on studies reporting *p*-values. Studies that measured outcomes of interest and found that the change in result values from pre- to post-intervention had a *p*-value of < 0.05 were classified as statistically significant. Studies with a change in result values that measured a *p*-value of ≥0.05 were classified as non-statistically significant. Studies that did not measure *p*-value were classified as no significance testing performed.

## Results

The updated search from 2014 to 2017 identified a total of 487 potentially relevant citations, and of these, 411 abstracts were excluded based on previously defined exclusion criteria. A total of 76 papers were selected for full-review, of which 44 studies met the inclusion criteria. Furthermore, 8 articles were included from supplemental searching. In parallel to the updated search, the full-texts of all 99 studies included in Dik et al. review were evaluated, of which 94 studies met our inclusion/exclusion criteria. As the primary objective of the Dik et al. review was to evaluate only the quality of these studies, we worked to synthesize the quantitative results of all these studies bringing the total count to 146 primary research studies for final analysis from 2000 to 2017 (Fig. 1).

### General characteristics

The majority of the studies analyzed were conducted in North America (49%) and Europe (25%) with approximately two-thirds of the included articles being published within the last 5 years. Most studies followed a quasi-experimental study design in medium-large hospitals with 500–1000 beds (Table 1). Most studies implemented an “audit of and/or feedback on the antimicrobial therapy provided” as an intervention strategy (57%), followed by “altered therapy guidelines” i.e., creation of hospital treatment guidelines specific to combating AMR or the alteration of previous antimicrobial therapy guidelines (25%) (Table 2). Even though we didn’t identify studies that exclusively assessed behavioral change therapy, studies with similar elements were included in “therapy feedback” and “giving education”.

**Table 1:** General characteristics of the reviewed studies

Characteristic	Number	Percentage
Geography (*N* = 146)		
North America	72	49
South America	5	3
Europe	37	25
Asia	20	14
Africa	4	3
Middle East	3	2
Australia	4	3
Multi-Region	1	1
Publication Year (*N* = 146)		
2000–2002	8	5
2003–2005	14	10
2006–2008	16	11
2009–2011	11	8
2012–2014	51	35
2015–2017	46	32
Study Design (*N* = 146)		
ITS	16	11
Quasi-experimental study	77	53
Retrospective evaluation	11	8
RCT	14	10
Cost-analysis	10	7
Cross-sectional survey	2	1
Observational study	15	10
Unclear	1	1
Number of Beds in Hospital (*N* = 146)		
< 150	13	9
150–500	28	19
500–1000	41	28
> 1000	26	18
Unclear	38	26
Number of Patients Included (*N* = 146)		
< 100	12	8
100–250	20	14
250–500	17	12
500–1000	5	3
1000–1500	8	5
> 1500	21	14
Unclear	63	43

*Abbreviations*: *ITS* Interrupted time series, *RCT* Randomized controlled trials

**Table 2:** *Types of interventions* and outcomes

	Number of studies	Percentage
Intervention (*N* = 145)		
Therapy evaluation, review and/or feedback	82	57
Altered therapy guidelines	37	25
Giving education	18	12
Antibiotic restriction lists of pre-authorization	15	10
Rapid diagnostic tools	11	8
New biomarkers	4	3
Pre-analytic consultations	2	1
Antibiotic cycling	1	1
Other	1	1
Outcome Measures (*N* = 146)		
Antibiotic Use		
Antimicrobial resistance	22	15
Antibiotic usage	100	68
Patient Outcomes		
LOS (days)	79	54
Mortality rate	58	40
Overall readmission rate	23	16
o 28/30-day readmission rate	15	10
Economic Outcomes		
Antimicrobial costs	91	62
Implementation costs	9	6
LOS costs	3	2
Operational costs	22	15
Cost savings	54	37
Other^a^	47	32

*Abbreviations*: *LOS* Length of stay

^a^Other outcome measures include but are not limited to mechanical ventilator use, adherence to guidelines, QALYs, avoidable hospital cost, etc.

Outcomes from the interventions generally fell into three categories: antibiotic use, patient outcomes, and economic outcomes. The majority of the studies primarily reported antibiotic outcomes, with 68% of studies reporting changes in antibiotic usage. The most commonly reported patient outcomes were LOS and mortality within the hospital. For economic outcomes, 62% reported changes in antibiotic expenditure and 37% studies reported overall cost-savings (Table 2).

### Antimicrobial outcomes

#### Antimicrobial usage

Of the 100 studies that reported antibiotic usage, 80 studies reported 108 relevant outcomes related to antibiotic usage [8–87]. Statistical significance testing was performeded in 69% of these outcomes. Outcomes measured included changes in drug dosage, including defined daily dose (DDD), of certain antibiotics, changes in duration of antimicrobial therapy, including days of therapy (DOT), and proportion of patients on antimicrobial treatment. Most studies that measured antibiotic usage were conducted in medium-large sized hospitals with 500–1000 beds (26%).

**Fig. 2:**
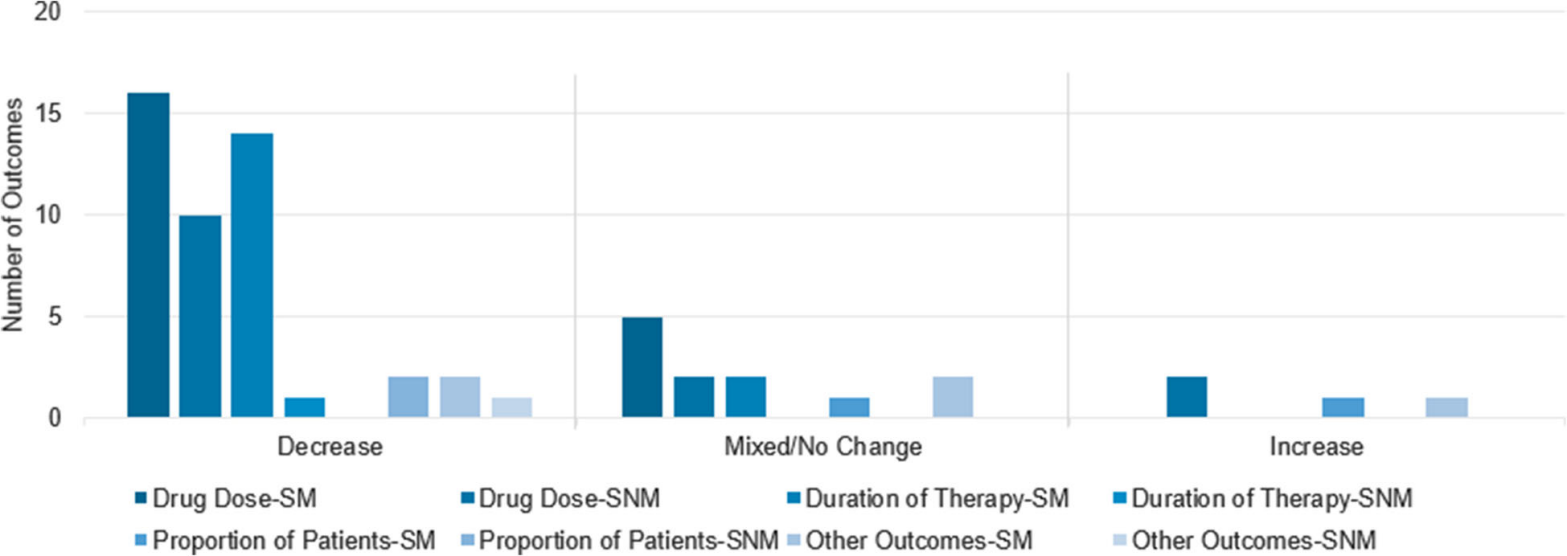
Effect of ASP on Total Antimicrobial Usage. SM = Significance measured; SNM = significance not measured. *Total usage of antibiotics decreased in a majority of studies, as measured by drug dose, duration of therapy, proportion of patients receiving antibiotic therapy, or other outcomes

Total usage of antibiotics decreased in majority of the studies, as measured by drug dose, duration of therapy, proportion of patients receiving antibiotic therapy, or other outcomes (Fig. 2). Usage of individual antibiotic classes (i.e., vancomycin, fluoroquinolones) demonstrated mixed trends. In most studies, use of some antimicrobials decreased, while use of other antimicrobials increased or did not change.

#### Antimicrobial resistance

Eighteen of 22 studies measured relevant outcomes related to AMR [7, 10, 11, 17, 19, 21, 23, 33, 34, 43, 63–65, 68, 82, 88–90]. Of these studies, 11 (61%) found a statistically significant change in AMR following implementation of a hospital ASP. These studies measured resistance after a mean control period of 21.2 months (range: 6 months–36 months) and change in resistance after a mean intervention period of 24.5 months (range: 6 months–36 months). Half the studies demonstrated a decrease in resistance for at least one microbial strain against an antimicrobial; two (11%) studies demonstrated a decrease in resistance for at least one antimicrobial coupled with an increase in resistance towards a different antimicrobial. Such instances can occur in an intervention strategy where the favored use of a certain class of antibiotics may increase selection pressure towards resistance. There was no obvious correlation between primary intervention strategy utilized and resistance, although the use of therapy evaluation, review, and/or feedback was the strategy utilized in both studies that reported both significant increases and decreases in antimicrobial resistance between different bacterial strains.

### Patient outcomes

#### LOS

Of the 79 studies reporting LOS, 68 measured 93 pertinent LOS outcomes [7, 8, 11, 17, 21, 24–26, 29, 31–33, 35–38, 40, 43–48, 50, 52, 56, 63, 66, 70, 72–79, 81–84, 89, 91–116]. Most of these studies occurred in medium-large sized hospitals with 500–1000 beds (32%).

The majority of the 68 studies (85%) reported either a reduction or no change in LOS that ranged from a decrease of 0 to 22 days (Table 3) after implementation of ASP. Only 10 studies reported an increase in LOS post-ASP implementation with a maximum increase of 5 days. More than half of the 93 measured outcomes (53%) did not show a statistically significant change; however, of the 33 outcomes that did reach statistical significance, approximately 88% showed a decrease in overall LOS. Studies of statistical significance showed an average decrease in LOS of 3.24 days or 20.6% per patient following ASP intervention.

**Table 3:** Literature synthesis of key outcomes: results and ranges

	# Studies Reporting Reductions or No Change	Range	# Studies Reporting Increases	Range
Patient Outcomes				
LOS	58	−21.9 to 0 days	10	0.1 to 5 days
All-cause mortality rate	41	−18.1 to 0%	13	0.02 to 11%
Infection-related mortality rate^a^	9	−12.0 to 0%	3	1 to 2.9%
All-cause readmission rate	13	−12 to 0%	8	0.2 to 8.6%
o 28/30-day	9	−10.86 to 0%	5	0.2 to 8.6%
Infection-related readmission rate	8	−2.94% to − 0.8%	2	0.3 to 0.65%
o 28/30-day	7	−2.94% to −0.7%	1	0.65%
Cost Outcomes				
Implementation costs	0	N/A	9	$2.5 k to $39.9 k
Annual operational costs^a^	11	−72.4% to − 12.9%	5	7.9 to 243%
Antibiotic costs	80	−80.1% to − 0.06%	7	4.1 to 51.5%
LOS costs^b^	2	-$18.3 k to -$1.95 M	0	N/A
Overall hospital costs^b^	32	-$9.11 k to -$2.06 M	0	N/A

^a^In these rows, the studies in the 2 columns are not mutually exclusive since more than 1 outcome was evaluated

^b^Only included studies measuring cost outcomes in USDN/A = Not Applicable

#### Mortality rate

Most studies that evaluated mortality rate occurred in medium-large sized hospitals with 500–1000 beds. Of 58 studies reporting mortality rate, 57 identified 73 relevant outcomes related to mortality rate. Among those studies, 54 studies reported 58 all-cause mortality rate outcomes [7, 11, 15, 19–22, 27, 28, 31–33, 35, 38, 40, 44–48, 50–52, 59, 62, 70, 72, 73, 75–82, 85, 91, 92, 95, 96, 98, 99, 102, 104, 105, 109, 110, 112, 113, 116–119]. Additionally, only 11 of the 57 relevant studies reported 15 infection-related mortality outcomes [7, 20, 48, 57, 74, 78, 79, 81, 83, 99, 109]. Statistical significance testing was performeded in 91% of these outcomes.

Of the 54 studies that reported changes in all-cause mortality, 77.2% reported reductions or no changes in mortality ranging from 0 to 18.1% decrease (Table 3). Similarly, the majority of the studies reporting infection-related mortality also showed reductions or no changes in mortality ranging from 0 to 12% decrease. Of the 58 all-cause mortality outcomes measured, 74% did not reach statistical significance. Of the 10 measurements that showed significance, 90% showed a decrease in mortality rate. Among studies that reported significant changes, there was an average decrease of 10.5% in all-cause mortality rates and 11.3% decrease in infection-related mortality rates following an ASP intervention.

#### Readmission rate

Twenty-three studies measured outcomes relevant to hospital readmission rate. 21 studies measured 24 relevant outcomes related to all-cause hospital readmission rate [15, 22, 29, 35, 38, 44, 48, 50, 51, 70, 72, 77–79, 83, 95, 105, 108, 109, 111, 118] and 10 studies measured 12 outcomes related to infection relapse readmission [38, 48, 77–79, 82, 105, 109, 111, 113]. Most of these studies occurred in medium-large sized hospitals with 500–1000 beds. Additionally, while there was variety in timepoint chosen for measuring readmission rate amongst the studies (range: 48 h to 90 days), most studies measured readmission rate at 28 or 30 days [15, 22, 35, 44, 50, 51, 77–79, 82, 95, 109, 111, 113, 118].

Of the 21 studies that evaluated all-cause readmission rates, 13 reported reduction or no changes ranging from 0 to 12%. Eight studies reported an increase in all-cause readmission with a maximum increase of 8.6% (Table 3). Of the 24 all-cause readmission outcomes, 86% did not reach statistical significance. Outcomes of statistical significance (*n* = 2) showed an average increase of 4.2% in the all-cause readmission rate following ASP intervention. Among the 12 infection-related readmission outcomes, 33% reached statistical significance and all showed a decrease in readmission rates (Table 3). Outcomes of statistical significance (*n* = 4) showed an average decrease of 1.2% in the infection-related readmission rate following ASP intervention.

### Economic outcomes

#### Implementation costs

Only 9 studies measured the cost of implementing an ASP [9, 58, 68, 106, 114, 118, 120–122]. Overall implementation costs did not seem to be associated with the type of ASP intervention (Table 3).

#### Operational costs

Operational costs were defined as the total direct hospital costs associated with patient treatment for bacterial infection, typically including costs associated with LOS, diagnostics, and treatment. A few studies also measured costs associated with human resources. All included studies measured costs from the hospital perspective and reported either total hospital costs or per patient costs both pre- and post-intervention. Of 22 studies, 17 measured 20 relevant operational cost outcomes, however only 13 studies including 16 of those outcomes measured the change in operational costs from pre- to post-ASP intervention [7, 25, 56, 70, 86, 92, 93, 100–103, 112, 123]. Most of these studies were from large hospitals with > 1000 beds (40%). Rapid diagnostic tools were the most utilized intervention strategy.

Operational costs varied in if they increased or decreased following ASP implementation, with 69% of outcomes demonstrating a decrease in annual costs and 31% showing an increase. Studies varied in reported currency so studies were only averaged together based on currency type due to wide discrepancies between currency conversion rates and years that studies were conducted. Of the 12 USD ($) studies, average ASP operational costs per patient were $5580 per year and increased by 5.97% between pre- and post- intervention, according to the 11 studies that measured those changes. However, there was a wide range in this change, with studies showing decreases in annual operational costs down to 72% or increases up to 236% (Table 3). Of the 4 EUR (€) studies, average ASP operational costs per patient were €1974.47 per year. Similar trends of increasing operational costs were exhibited amongst the 2 of 4 EUR (€) studies that measured change in cost with no studies exhibiting decreases in operational costs following ASP implementation (range: 7.93 to 243%, Table 3).

Changes in operational costs seemed to have a correlation with the intervention strategy utilized. Use of altered therapy guidelines and antibiotic restriction lists of pre-authorized agents typically reduced annual operational costs, on average of 17.1 and 17.5%, respectfully. Therapy evaluation, review, and/or feedback increased operational costs by an average of 27.5%. Some interventions that utilized rapid diagnostic testing or new biomarkers as their primary intervention strategy more than tripled their operational costs at increases of 236 and 243%, respectfully.

#### Antimicrobial expenditures

Of 91 studies, 87 measured 94 pertinent antimicrobial expenditure outcomes but only 46% of outcomes were assessed for statistical significance [8, 9, 12–22, 26, 28, 30–34, 37, 39–48, 52, 53, 55–58, 60, 62–68, 70–73, 75, 76, 82–84, 87–89, 91, 93, 95, 97, 99, 100, 104, 106, 107, 117, 118, 120, 122–138]. Most studies were conducted in hospitals with 500–1000 patients (26%). Approximately 92% of the 87 studies showed a decrease in antimicrobial costs ranging from 0.06 to 80.1%. Of the 94 outcomes, only 15% did not reach statistical significance compared to 31% that demonstrated significant change. Of the outcomes that demonstrated significant changes, 97% showed a decrease in antimicrobial costs, averaging 35.6% decrease in costs following ASP implementation. Only one study showed a significant increase of antimicrobial costs (51.5%) (Table 3).

#### LOS costs

All 3 studies that reported changes in LOS costs demonstrated decreases in costs following ASP implementation [43, 66, 118]. This ranged from decreases of $18,305 for a small hospital to $1.95 M (Table 3) and 970,397 kr (Swedish Krona/SEK) for two large sized hospitals. All studies utilized therapy evaluation, review, and/or feedback as their primary intervention strategy.

#### Overall cost savings

Of 54 studies, 49 studies measured 58 relevant overall cost savings outcomes [9–11, 23, 26, 27, 35, 36, 38, 42, 46, 49, 50, 52, 54, 57, 59, 60, 64, 76, 81, 82, 90, 91, 95, 96, 98, 99, 105, 106, 108, 112, 114, 117, 119, 120, 127–129, 134, 137, 139–146]. Most of these studies were conducted in medium sized hospitals with 150–500 beds (26%). Studies varied widely in their reported currency as well as annual cost savings, although most of the studies measured costs in US Dollars (65%). Average cost savings in US Dollar (USD) studies were $435,000 (range: $9110–$2.06 M) per year for the hospital (Table 3), or $732 (range: $2.50 -$2640) per patient. Average cost savings in Euro (EUR) studies were €41,500 (range: €19,000–€66,200) per year for the hospital, or €198.00 (range: €40.40–€529.00) per patient. Average cost savings in Great Britain Pound (GBP) studies were £144,000 (range: £7120–£286,000) per year for the hospital, or £304.00 (range: £2.47–£1000) per patient.

## Discussion

Through the implementation of a range of interventions, hospital ASPs aim to provide high value for patients, with value defined as the health outcomes achieved per dollar spent [147]. This goal unites the interests of all the stakeholders in the system, including patients. If value improves, patients, payers, providers, and suppliers can all benefit while the economic sustainability of the health care system increases. This approach to considering sustainable funding and prioritizing of ASP activity has been used successfully in a range of other disease areas such as cardiovascular disease, diabetes, musculoskeletal diseases, etc. [148].

Value in health care is measured by the outcomes achieved and not by the volume of services delivered thereby shifting focus from volume to value. Value is also not measured by the process of care used. Even though process measurement and improvement are important tactics they are not substitutes for measuring outcomes and costs. Cost reduction without regard to the outcomes achieved is dangerous and self-defeating, leading to false “savings” and potentially limiting effective care.

**Fig. 3:**
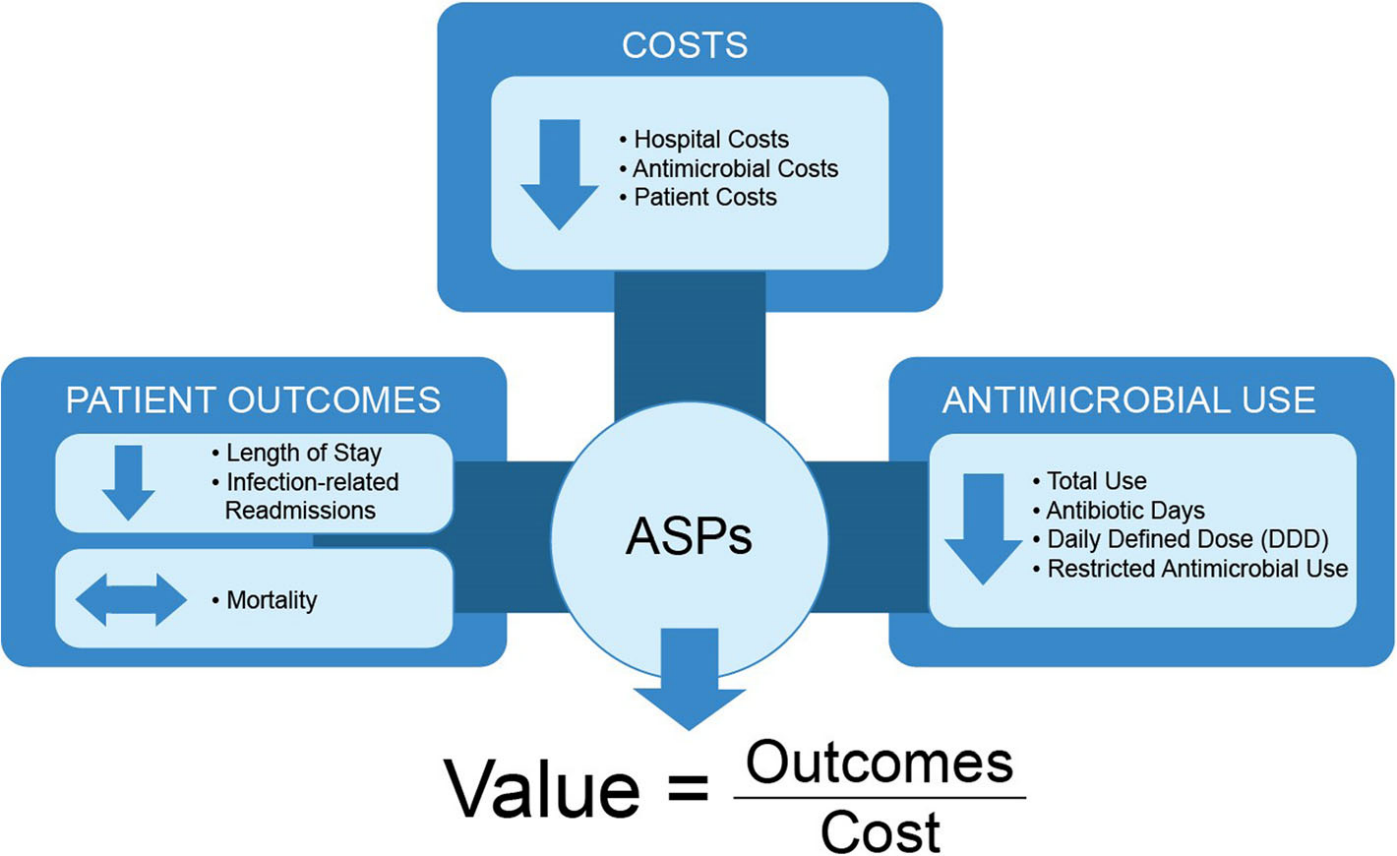
Conceptual value framework for implementation

Hospital ASPs aim to promote the efficient and judicious use of antimicrobials to combat the rise in AMR. While most ASPs focus on changes to antimicrobial use practices, the effects of ASPs often yield downstream effects that extend from antimicrobial use into improved or maintained patient outcomes (numerator of the value equation) which drive down resource utilization and associated costs (denominator of the value equation). To reduce cost, the best approach is often to spend more on some services to reduce the need for others. Despite some significant limitations [described later] of the available data, the majority of the studies included here appear to produce similar results in support of our value framework (Fig. 3), particularly in relation to ASPs in North America and Europe. However, there were a number of studies which reported contradicting results, such as an increase in the use of certain antibiotics, worsening of some patient outcomes, and increased hospital costs. It is important to try to understand potential reasons for this.

In relation to antibiotic use, a few studies demonstrated a significant increase in use of at least one antibiotic class [10, 31, 32, 72, 76]. This could be due to many reasons including an inappropriate metric for measuring antimicrobial use [76]. For example, an increase in use of narrow spectrum antibiotic use offsets a decrease in broad spectrum use yielding lower AMR [10, 31, 32, 72]. Additionally, if the consumption of only certain antibiotics is restricted, a global decrease in resistance cannot be expected [63]. It is possible that resistant strains are unrelated to changes made in hospitals, as there may be an inability to differentiate between community- or hospital-acquired isolates [17, 21].

Some studies found that patient LOS increased following ASP implementation [21, 36, 44–46, 70, 75, 82, 97, 102]. LOS can be influenced by factors beyond antibiotic use, including comorbidities and disease severity, yielding non-statistically significant increases in patient hospital days [21, 36, 44, 75, 82, 97, 102]. Targeted interventions (e.g., focus on reducing particular antibiotics) may not lead to overall changes in LOS [36, 97, 102]. Short study durations or changes in pre- and post-intervention populations may also compromise the direct results of the ASP on global LOS [45, 46, 75, 82, 97, 102]. Similarly, a few studies found that patient mortality increased following ASP implementation but majority of these studies did not show statistical significance [33, 45, 46, 70, 78, 79, 99, 102, 113]. Reasons could include short study durations [45, 82], presence of an existing ASP prior to the new intervention strategy that may have limited the ASP impact [82], delayed therapy due to unavailable diagnostic results [102, 113], or poor communication between ASP staff members regarding treatments [82, 113]. Differences in the pre- and post-ASP populations and poor communication between ASP staff members may be factors that led to an increase in readmission rates in some studies [72, 77].

The small number of economic outcome studies that do not show a significant reduction in antimicrobial costs following an ASP instead showed no significant changes or did not measure changes for significance [17, 28, 83, 97, 100]. Short study durations [97], increase in the use of higher cost antibiotics that are more effective in decreasing AMR [17, 41], and lack of adherence to ASP interventions by all hospital staff members [41] could lead to increase in antimicrobial costs.

Overall an increase in ASP costs may be offset by total cost savings for the hospital [70, 112]. In addition, at a per-patient level, the average per-patient cost savings represents a significant portion of a hospital bed-day cost. For example, in the EU and UK, the proportion of a bed day saved through ASP represents 60–80% of the cost of a bed day, while in the US the proportion of a bed day saved is lower (~ 32%). Reduced patient LOS is often the key driver of per patient cost savings for a hospital and may explain the significant difference in cost savings that were found between US, UK, and EU hospitals. As per Table 4, average higher per patient cost savings were realized in studies conducted in the US, largely due to the high cost of a hospital bed day in the country.

**Table 4:** Cost savings compared with bed day costs around the world

	United States	European Union	United Kingdom
Annual Per Patient Cost Savings with ASP	$732.00	€198.00	£304.00
Average Hospital Bed Day Cost, 2015	$2271 [2]	€328.64 [154, 155]^a^	£375.86 [154, 155]^a^
Estimated Cost Offset as a Bed Day Saved Annually	32%	60%	80%

^a^Original WHO 2008 costs in I$ were inflated to 2015 costs and converted to Euro or Pound Sterling

There were some limitations associated with the studies included in this review. First, there was heterogeneity among the studies, in terms of size of hospitals and patient populations included in the review, as previously described in Table 1. Some studies implemented ASPs across the entire hospital with patient pools of nearly 13,000 [43], whereas other hospitals only implemented their programs in specific wards or units with < 500 patients [75, 106]. Furthermore, numerous studies did not indicate the size of their hospital bed size, patient population, or both, making it challenging to compare their results to either smaller medical centers with < 100 beds [15] or large hospitals with > 600 beds [43]. The geographic distribution and provision of healthcare delivery is biased towards the North American and European hospital setting thereby rendering it difficult to apply these findings globally. This challenge is further driven by the wide range in years that the studies were conducted as well as their reported currency, preventing a comparison of the economic results on the same currency and same inflation rate. Homogenizing the currency and year of the studies would have allowed us to conduct more analysis into the effectiveness of different ASP strategies and program designs to draw conclusions on why certain programs drove improved outcomes and cost savings. Additionally, the studies varied in the type of ASPs, outcomes and follow-up periods. Despite this limitation, our review was not restrictive in the studies included. Second, the majority of the studies did not report start-up and implementation costs. The main economic outcome of interest was costs associated with antimicrobial use. More well-designed studies are needed that will evaluate the costs of implementing ASPs to truly capture the economic burden.

This is the first systematic review that provided a comprehensive summary of results of the economic impact of ASPs. Given our objective to conduct a broad review of the financial literature evaluating ASPs, the search was not restricted to only include certain study designs, interventions or study durations. A future step would be to conduct a meta-analysis of the results with restrictive inclusion/exclusion criteria to further delve into the relationships between specific ASP interventions and economic outcomes.

## Conclusions

Overall this systematic review demonstrates that ASPs can offset or reduce costs while improving some patient outcomes, thereby suggesting high value for certain healthcare systems. The findings also suggest that costs associated with start-up and implementation of ASPs are potentially offset by subsequent cost-savings. Additionally, numerous systematic reviews and meta-analyses have demonstrated that such programs have beneficial effects on hospital LOS [149, 150], resistance patterns [63, 150], and infection incidence [151]. This data supports the value of ASPs in tandem with infection control measures [1]. However, for the findings to be globally relevant, more studies, particularly in real world settings across a diverse range of geographies and resource settings are required, so that a full critical appraisal of the true value of these programs can be made. This will not only allow our ability to develop high value bespoke models of ASP based on robust clinical and economic data but also consider creating benchmarks, an area fraught with challenges [152, 153].

## Abbreviations

AMR: Antimicrobial resistance
ASP: Antimicrobial stewardship program
DDD: Daily defined dose
DOT: Days of therapy
EUR: Euro
GBP: Great Britain Pound
kr: Swedish Krona
LOS: Length of stay
PRISMA: Preferred Reporting Items for Systematic Reviews and Meta-Analyses
USD: United States Dollar
